# Ion-Doped Photonic Crystal Fiber Lasers

**DOI:** 10.3389/fchem.2021.801477

**Published:** 2021-12-24

**Authors:** Ya-Chong Hou, Yun-Fei Li, Xiao-Fan Xie, Zi-Long Kou, Yue Lu, Si-Ying Chen, Yulei Wang, Zhiwei Lu

**Affiliations:** ^1^ Center for Advanced Laser Technology, Hebei University of Technology, Tianjin, China; ^2^ Hebei Key Laboratory of Advanced Laser Technology and Equipment, Tianjin, China

**Keywords:** fiber, laser, photonic crystal, ion doping, output characteristics

## Abstract

Compared with conventional solid-state lasers, fiber lasers have the advantages of small size, simple cooling system, and good output beam quality, enabling them an extended service lifetime in industrialized environments. Periodically arranged photonic crystals have been the most important gain medium for high-power laser applications, which overcame the problems in fiber lasers such as small mode field, low degree of nonlinearity, and non-adjustable dispersion. In this mini-review, we summarize the recent advances of typical ion-doped photonic crystal fiber lasers doped, discuss the challenges, and provide an outlook on the future developments in ion-doped photonic crystal fiber lasers.

## Introduction

High-quality output laser systems are constantly pursued in solid-state lasers, due to their applications in laser ranging and laser medicine (Kim et al., 2020; Miura et al., 2020; Chen et al., 2021). However, traditional solid-state lasers have severe temperature effects and generate a lot of heat, influencing the output performance. Due to the flexible structure, large mode field area (Knight et al., 1998), photonic crystal fibers maintain the superior characteristics of infinite single mode (Birks et al., 1997), low loss and adjustable dispersion (Ferrando et al., 2000; Ortigosa-Blanch et al., 2000; Zhang, 2012), overcoming the defects of conventional fibers, and have replaced the traditional YAG. So far, photonic crystal fibers have great potential for applications in fiber lasers, fiber optic communication (Han, 2010), fiber optic sensing (Kang et al., 2015) and nonlinear optics (Dudley and Taylor, 2009), so researchers have extensively studied the doping of different ions in photonic crystal fibers, and thus prepared photonic crystal fiber lasers with different output characteristics.

Currently, the power enhancement of fiber lasers is mainly affected by nonlinear effects (NLEs) (Zervas and Codemard, 2014a; Man Hu et al., 2016), photodarkening (PD) effects (Jetschke et al., 2008), and Transverse mode instability (TMI) effects (Tao et al., 2015). Large mode field fibers are usually used instead of conventional small core diameter double cladding fibers to suppress the nonlinear effects and thus increase the output power. Although increasement of the core diameter is an effective method to achieve a large mode field, this will affect the beam quality. To resolve the conflict between large mode field and beam quality, the structural design of the fiber and rare earth ion doping can be employed to suppress the photodarkening effect of the fiber (Jauregui et al., 2015; Petit et al., 2016). One of the important strategies adopted to increase the mode instability threshold is the preparation of large mode field fibers with smaller core numerical apertures. These mentioned problems can be solved by the ion doping of photonic crystal structured fibers. However, the preparation of doped photonic crystal fibers need to meet the geometric size, optical (refractive index, homogeneity) and spectral (doping concentration, absorption and emission) performance requirements, posing a challenge for these techniques.

In this mini-review, we summarize the recent advances in doped photonic crystal fiber lasers, describe the basic structure and optical conduction mechanism of photonic crystal fibers. Several typical photonic crystal fiber lasers have been reviewed, including ytterbium ion-doped photonic crystal fiber lasers, erbium ion-doped photonic crystal fiber lasers, thulium ion-doped photonic crystal fiber lasers, and other doped types of photonic crystal fiber. In addition, the mini-review briefly discusses the challenges in the field and provides an outlook for the future.

## Structure and Light Conduction Mechanism of Photonic Crystal Fibers

Photonic crystal fiber (PCF), is also known as microstructured fiber (MOF) or porous fiber (PF). Since the first photonic crystal fiber was successfully developed by Knight group in 1996, photonic crystal fibers had been hotly pursued in laser systems for years. Photonic crystal fibers have been the most important gain medium for high-power laser applications, which possess flexible structure and high optical characteristics overwhelming traditional fiber structures (Cheng et al., 2014; Li et al., 2019a; Lv et al., 2020a; Li et al., 2020). Photonic crystal fibers can be divided into total internal reflection photonic crystal fibers, photonic bandgap photonic crystal fibers, and anti-resonant photonic crystal fibers based on transmission mechanisms. Besides, they can also be divided into solid-core photonic crystal fibers and hollow-core photonic crystal fibers based on structures (Knight, 2003; Russell, 2003; Belardi and Knight, 2014). Among them, solid-core photonic crystal fiber, also known as refractive index-guided fiber, is composed of a fiber core and a cladding consisting of periodically arranged air holes around it. The light-guiding mechanism of such fiber is total internal reflection (Markos et al., 2017). Subsequently, solid-core photonic crystal fibers gradually evolve into nonlinear photonic crystal fibers, large-mode field photonic crystal fibers, and rare-earth-doped photonic crystal fibers with different structures and functions.

Hollow-core photonic crystal fibers can be divided into two types by light conduction mechanisms, namely hollow-core photonic bandgap, and hollow-core anti-resonant. The core of hollow-core photonic bandgap photonic crystal fibers is generally filled with air or other gases, whose light conduction mechanism is photonic bandgap effect, as opposed to the total internal reflection type fibers (Huang et al., 2017; Boufenar et al., 2018). In a photonic bandgap fiber, periodic structure can be formed by alternating arrangements of high and low refractive index materials, which influences the photonic bandgap. The distances between the periodically low refractive index sites are the same, resulting in the confinement of light only for a certain frequency of light in the core. Based on the band gap effect, light is only transmitted in a low refractive index core, which greatly reduces transmission losses. Moreover, the core can be adjusted according to the actual situation, which can be 3 cores, 7 cores, and other multi-core structures (Zhu et al., 2012; Xiang and Jiang, 2018; Lv et al., 2020b). The other kind is anti-resonant hollow-core photonic crystal fiber (Markos et al., 2015), whose cladding consists of only one layer of air holes. The light conduction mechanism of this fiber is different from that of hollow-core bandgap fiber; and the structure can be regarded as a resonant cavity, due to the high refractive index region on the inner wall of the cladding. In the resonant state, light leaks out of the cladding since the resonant cavity can be regarded as transparent. In the non-resonant state, the reflection coefficient of the resonant cavity has a high reflection coefficient, which limits the propagation of light within the core (Kudlinski et al., 2020). It is the flexibility and versatility of the photonic crystal fiber structure improves the output characteristics of lasers, which gives it advantages than the conventional fiber structure.

## Ion-Doped Photonic Crystal Fiber Lasers

**FIGURE 1:**
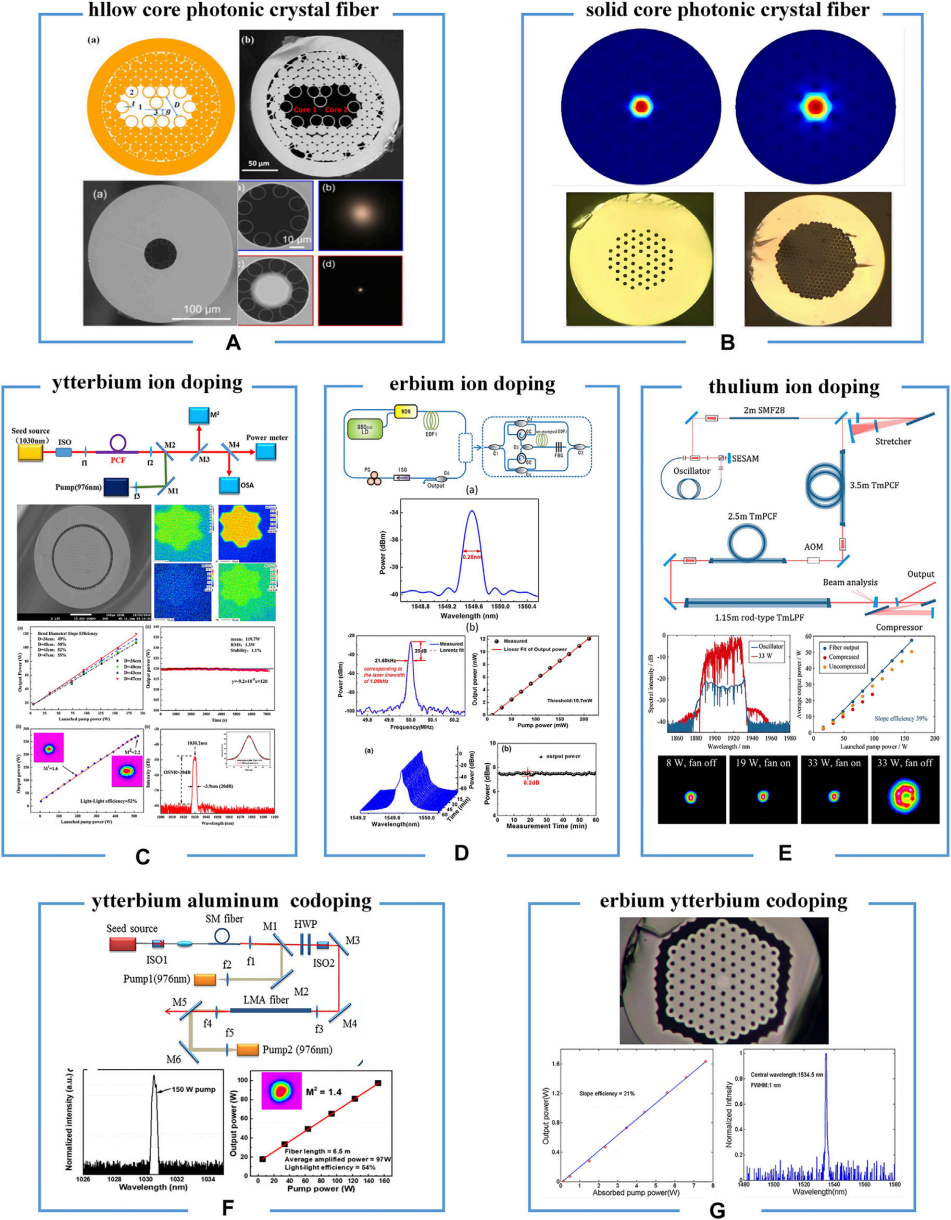
Ion-doped photonic crystal fiber lasers. **(A,B)** Types of photonic crystal fibers. **(C–G)** Various ion-doped photonic crystal fiber lasers such as Ytterbium ion doping **(C)**, Erbium ion doping **(D)**, Thulium ion doping **(E)**, Ytterbium aluminum codoping **(F)**, and Erbium ytterbium codoping **(G)**.

It is the unique advantages of photonic crystal structured fibers have stimulated research interest in the development of special lasers with various outputs. In this section, we briefly summarize the recent advances of representative ion-doped photonic crystal fiber lasers (Figure 1).

## Ytterbium-Doped Ions

In 2012, IPG achieved 10 kW single-mode fiber laser output using a large-mode-field double-clad fiber with a core diameter of about 25 μm, which became a milestone in the development of photonic crystal fiber lasers (Limpert et al., 2012). Inspired by IPG, researchers have developed a variety of photonic crystal fiber lasers. As the photodarkening effect of the fiber can be improved by doping with ions, the photonic crystal fiber structure has large-mode field properties that can withstand high power without optical damage and ensure good beam quality. Ion-doped large-mode field photonic crystal fibers have attracted significant attentions in high peak power picosecond ultrafast laser amplifiers (Zervas and Codemard, 2014b; Diouf et al., 2016; Kabir and Razzak, 2018; Li et al., 2019b). The most common doping ion in photonic crystal fiber is ytterbium ion. Specifically, Otto et al. (2014) obtained an average output power of 2 kW in an ytterbium-doped large-span photonic crystal fiber, including a peak power of 0.8 MW with a beam quality M^2^ less than 3 (Wei et al., 2016). Wei et al. (2016) investigate an all-solid-state large-area ytterbium-doped quartz photonic crystal fiber, in which they achieved an all-solid-state large-mode field microstructured fiber with a core diameter of 50 μm. The fiber laser could maintain quasi-single-mode transmission characteristics in 1,064 nm and obtain a laser output of 8 W. In addition, Wang et al. (2019) also successfully developed a quasi-single-mode picosecond pulsed laser with ytterbium-doped large-mode field photonic crystal fiber, in which the inner cladding diameter was about 260 μm with outer diameter of about 450 μm, air hole diameter of about 2.5 μm, and the spacing of about 14 μm. At the optimal bending diameter of 47 cm and 520 W pumping power, the fiber laser achieved a maximum output power of 272 W, a single pulse energy of 5.6 μJ, and a peak power of 266 kW. However, the beam quality gradually deteriorated with the increased power, owing to the thermal accumulation of the core. The thermal accumulation triggered the change in the refractive index of the core, leading to a mismatch in the numerical aperture of the core and further affecting the laser beam quality (Li et al., 2010). Subsequently, Wang et al. further prepared an ytterbium-doped large-mode field photonic crystal fiber laser with a core diameter of 75 μm, based on an optimized fabrication process, in which the air aperture was approximately 2 μm and the spacing was approximately 16 μm. The corresponding fiber laser obtained an average output power of 102 W with a single pulse energy of 102 μJ and a peak power of more than 1 MW at 166 W pumping power. This experiment had not observed excited stimulated Raman scattering (SRS) emission phenomenon. The Yb-doped large-mode field photonic crystal fiber lasers improved greatly in terms of picosecond pulsed laser amplification performance with the improvement of the Yb-doped core material preparation technology.

## Erbium-Doped Ions

Although ytterbium-doped ion photonic crystal fibers are still the mainstream of laser development, erbium-doped ion photonic crystal fiber lasers have also been extensively studied in recent years, especially in narrow linewidth output with ultrashort pulse lasers (Li et al., 2015). Li et al. (2015) obtained a three-wavelength narrow linewidth output of less than 2 kW or a six-wavelength narrow linewidth output of less than 5 kW using a tunable variable optical attenuator based on Rayleigh scattering for tunable narrow linewidth Brillouin-doped photonic crystal fiber lasers (Pourshab et al., 2017). Pourshab et al. (2017) obtained the laser output at 1,561.47 nm with an uplink linewidth of less than 1.19 kHz using an erbium-gallium co-doped photonic crystal fiber as the gain medium and a narrow-band filter, a sub-ring cavity and a cascaded anisotropic fiber as mode selection. Narrow linewidth lasers are important in a variety of fields where high precision and strong coherence are required. In addition, Elahi et al. (2017) overcame the spectral narrowing effect of erbium-doped photonic crystal fiber in the amplification process and obtained an ultrashort pulse output with a minimum pulse width of 175 fs, a repetition frequency of 43 MHz, a central wavelength of 1,550 nm, and a single pulse energy of 80 nJ in an erbium-doped photonic crystal fiber pulse amplification system.

## Thulium-Doped Ions

At present, there are fewer reports on Nd-doped photonic crystal fiber lasers compared to Yb-doped photonic crystal fiber lasers and Erbium-doped photonic crystal fiber lasers. A chirped amplification system using a thulium-doped photonic crystal fiber with a mode field diameter of 65 μm and an air cladding of 260 μm was established by Stutzki et al. (2015) A pulsed peak power of 200 MW, an average output power of 24 W, and a pulsed energy of 120 μJ were achieved with a 790 nm laser diode backward pumping. In addition, in their study of compact thulium-doped photonic crystal fiber lasers, Lee et al. (2020) achieved a picosecond pulse output in 2 μm with an average output power of up to 25 W. This pulse was compressed by a compact multipass configuration, resulting in a maximum output pulse energy of 46.3 μJ and a maximum output pulse width of 2.8 ps.

### Other Photonic Crystal Fiber Lasers

In recent years, several researchers have used chemical vapor deposition techniques to prepare photonic crystal fibers with very low numerical apertures using various co-doping ions such as Yb^3+^/Al^3+^/P^5+^ and Yb^3+^/Al^3+^/F^−^. For example, in their study of large-mode field photonic crystal fiber lasers, Wang et al. (2017) successfully prepared a fiber with a core diameter of 50 μm using Yb^3+^/Al^3+^/P^5+^/F^−^ co-doping and achieved a laser output with an average power of 97 W, a peak power of 93 kW, and a beam quality of 1.4. Since high-power fiber lasers in the 1.5 µm band have a wide range of applications and the gain medium for fiber lasers in this band is usually a double-clad erbium-ytterbium co-doped fiber, so that ytterbium-erbium co-doped photonic crystal fibers dominate among other dopant ions, Of which Wang et al. (2014) studied large-mode field fiber lasers using erbium-ytterbium co-doping in a stable laser output with a maximum power of 1.6 W and a center wavelength of 1,534.5 nm obtained in a 600 mm length photonic crystal fiber. The pump optical power was 150 mW, with the slope efficiency of 21%, and the beam quality factor M^2^ less than 1.05. The co-doped photonic crystal fiber has excellent laser performance and is an important direction for future development in fiber lasers.

## Conclusion and Outlook

In summary, optical fibers with photonic crystal structures are flexible and versatile, featuring large mode field area, infinite single mode, low loss, and hold great promise for the preparation of high-power ultrashort pulse lasers. This mini-review summarizes the recent advances in photonic crystal fiber lasers doped with different ions, including ytterbium-doped photonic crystal fiber lasers, erbium-doped photonic crystal fiber lasers, thulium-doped photonic crystal fiber lasers, and other types of photonic crystal fiber lasers. However, due to the special nature of photonic crystal structures, the major challenge at present is that the pulling of photonic crystal fibers. 3D printing microstructured optical fiber process is an effective approach to solve the problem of difficult preparation of microstructured fiber prefabricated rods and long-distance fiber drawing, paving a way to the future development of photonic crystal fiber. In addition, Compared to photonic crystal structured lasers and sensors, photonic crystal structured optical fibre communication has been less researched, but there is great potential for development due to its unique properties such as photonic band gap effect, unique waveguide properties and non-linearity making it important in areas such as optical wave transmission and control, optical information manipulation and processing and new photonic devices. In conclusion, compared with conventional optical fibers, photonic crystal structured optical fibers possess significant research values in numerous fields.
